# Is there an association between quadriceps thickness and functional capacity in patients with chronic kidney disease?

**DOI:** 10.1590/2175-8239-JBN-2021-0274

**Published:** 2022-04-13

**Authors:** Bruno Lionardo de Paula, Bruno Valle Pinheiro, Dilmerson de Oliveira, Maycon Moura Reboredo

**Affiliations:** 1 Universidade Federal de Juiz de Fora, Núcleo de Pesquisa em Pneumologia e Terapia Intensiva, Juiz de Fora, MG, Brasil.; 2 Universidade Federal de Juiz de Fora, Faculdade de Medicina, Juiz de Fora, MG, Brasil.; 3 Centro Universitário FAMINAS, Muriaé, MG, Brasil.

Dear Editor:

In a recently published paper, Costa et al.^1^ demonstrated the association between ultrasound-assessed quadriceps muscle thickness and the number of repetitions in the 60s sit-to-stand (STS) test (R^2^ = 0.436; p = 0.037) in patients with pre-dialysis chronic kidney disease (CKD). However, quadriceps thickness was not associated with the distance covered in the six-minute walk test (6MWT) and with handgrip strength^1^. Therefore, this letter aims to discuss some findings of the study by Costa et al.^1^, considering our experience with the STS test and the partial results of a project that we are carrying out to evaluate the association of different STS test protocols with the quadriceps muscle torque in patients with CKD on hemodialysis.

In our study, the patients underwent three STS protocols (5 repetitions, 10 repetitions and 30s) and quadriceps muscle torque assessment in the dominant lower limb by manual dynamometry (MMT Lafayette Instrument Company, USA). Forty patients have been included so far (24 women; 59.7±11.9 years; 26.6±5.3 kg/m^2^). Multiple linear regression models were created to assess the association between STS test results (dependent variable) with muscle torque, adjusted for age, sex, and body mass index. The quadriceps muscle torque was associated with the 5-repetition STS tests (R^2^ = 0.434; B =-0.112; CI =-0.163,-0.061; p < 0.001), 10-repetition STS (R^2^ = 0.545; B =-0.245 ; IC =-0.348,-0.142; p < 0.001) and 30s STS (R^2^ = 0.508; B = 0.110; IC = 0.066, 0.153; p < 0.001). These partial results suggest that the 10-repetition STS test represented the best strategy to estimate muscle torque in hemodialysis patients. The 60s STS test was not applied to our patients, because this protocol is more associated with resistance and not with muscle torque/strength^2-5^.

Although the results by Costa et al.^1^ showed that the ultrasonography-assessed quadriceps muscle thickness was associated with performance in the 60s STS test, this relationship was not seen with distance in the 6MWT. On the other hand, some studies have shown that there is an association between the 60s STS test result and distance in the 6MWT; and have also described that the 60s STS test may represent an alternative to determine functional capacity, especially if space and time are limited^2-5^. Thus, we believe that the results found by Costa et al.^1^ are actually more associated with the characteristics of the 60s STS test in assessing muscle endurance than the relationship with the functional capacity in patients with CKD. Basically, ultrasonography enabled us to analyze muscle “volume” rather than muscle “quality” by determining fiber types in the quadriceps. This fact may also explain the lack of association between quadriceps thickness and handgrip strength.

Therefore, despite the importance of using ultrasound for muscle assessment in patients with CKD, functional tests such as the 60-s STS and the 10-repetition STS may better represent muscle endurance and strength, respectively.
